# Restrictive vs. liberal blood transfusion strategies in Africa: clinical outcomes, resource stewardship, and future directions

**DOI:** 10.3389/fmed.2026.1913287

**Published:** 2026-09-28

**Authors:** Emmanuel Ifeanyi Obeagu

**Affiliations:** 1 Division of Haematology, Department of Biomedical and Laboratory Science, Africa University, Mutare, Zimbabwe; 2 Department of Molecular Medicine and Haematology, School of Pathology, Faculty of Health Sciences, University of the Witwatersrand, Johannesburg, South Africa

**Keywords:** Africa, blood transfusion, liberal transfusion, patient blood management, restrictive transfusion

## Abstract

Blood transfusion continues to be a vital part of contemporary healthcare, especially in treating severe anemia, bleeding, trauma, obstetric crises, and critical conditions. In Africa, where transfusion-dependent conditions are prevalent and blood supplies often limited, establishing the best transfusion threshold is crucial for enhancing patient outcomes while maximizing the use of scarce resources. Restrictive transfusion practices that typically use lower hemoglobin thresholds (7–8 g/dL) are now widely accepted globally as they minimize unnecessary transfusions while maintaining safety across various clinical environments. In contrast, liberal approaches, defined by elevated transfusion thresholds (9–10 g/dL), might still be suitable for specific patient groups, such as individuals with acute coronary syndromes, persistent significant bleeding, or serious symptomatic anemia. This review analyzes the existing evidence regarding restrictive vs. liberal blood transfusion strategies in the African context, focusing on clinical outcomes, resource management in healthcare, and prospective avenues for practice and investigation. Research from randomized controlled trials, observational studies, and global guidelines consistently shows that restrictive transfusion strategies correlate with similar mortality, morbidity, hospital stay duration, and functional recovery in most stable hospitalized patients, while minimizing transfusion-related complications and preserving limited blood resources. Nevertheless, the direct relevance of evidence produced mainly in high-income countries needs thorough evaluation due to Africa's distinct epidemiological characteristics, healthcare system constraints, and blood service deficiencies. Enhancing patient blood management initiatives, increasing voluntary blood donations, improving transfusion oversight, and producing robust African clinical evidence are essential priorities.

## Introduction

Blood transfusion is a life-saving intervention for severe anemia, acute blood loss, and selected hematological and critical illnesses. Across Africa, however, transfusion services operate within a challenging environment characterized by a high burden of anemia, obstetric hemorrhage, trauma, malaria, sickle cell disease, infectious diseases, and chronic disorders, alongside persistent variability in blood availability, laboratory capacity, component therapy, and access to emergency care. These factors make decisions about when and how much blood to transfuse particularly important (1–3). Historically, liberal transfusion practices favored maintaining relatively high hemoglobin concentrations, based on the assumption that increased oxygen-carrying capacity would improve tissue oxygenation and clinical outcomes. Increasing evidence from randomized trials and systematic reviews has challenged this approach, demonstrating that restrictive transfusion strategies, generally using lower hemoglobin thresholds, are safe for many clinically stable, non-bleeding patients and substantially reduce exposure to allogeneic blood. Nevertheless, transfusion thresholds are not universally applicable. Patients with active hemorrhage, severe symptomatic anemia, selected cardiovascular conditions, and certain hematological or pediatric disorders may require individualized or more liberal approaches (4–7).

The African context adds an important dimension to this debate. Much of the evidence underpinning contemporary transfusion thresholds originates from high-income countries, whereas African populations experience different disease patterns, baseline anemia, healthcare infrastructure, blood-supply constraints, and delays in accessing definitive care. Consequently, international transfusion thresholds cannot always be applied without considering local clinical and health-system circumstances (8, 9). Although previous reviews have extensively examined restrictive and liberal transfusion strategies, comparatively less attention has been given to their integration with African blood-resource stewardship and Patient Blood Management (PBM). The limited availability of African randomized evidence further creates uncertainty regarding the generalizability of established transfusion thresholds across the continent (10, 11). This narrative review therefore examines the clinical outcomes, physiological considerations, resource implications, and implementation challenges associated with restrictive and liberal blood transfusion strategies, with particular emphasis on African healthcare settings. It also evaluates evidence in special populations and identifies priorities for African-specific research, PBM implementation, and development of context-sensitive transfusion policies.

### Objectives

The overall objective of this narrative review is to critically examine restrictive vs. liberal blood transfusion strategies in Africa, with emphasis on clinical outcomes, patient safety, blood-resource stewardship, and applicability to diverse African healthcare settings.

The specific objectives are to:

1. Examine the physiological and clinical rationale underlying restrictive and liberal red-cell transfusion strategies.
2. Compare clinical outcomes associated with restrictive and liberal transfusion approaches, including mortality, morbidity, organ dysfunction, transfusion-related complications, and healthcare utilization.
3. Evaluate evidence from major randomized controlled trials and evidence syntheses that have shaped contemporary transfusion thresholds and recommendations.
4. Assess the applicability of international transfusion evidence to African populations, considering differences in disease burden, baseline anemia, healthcare infrastructure, blood availability, and access to emergency care.
5. Examine transfusion strategies in important African clinical populations, including children with severe anemia, pregnant women with obstetric hemorrhage, trauma patients, critically ill patients, individuals with sickle cell disease, and patients with hematological malignancies or chronic transfusion requirements.
6. Evaluate the implications of restrictive and liberal transfusion strategies for blood availability and resource stewardship, particularly in settings where blood supplies and transfusion infrastructure are limited.
7. Examine the role of Patient Blood Management (PBM) in reducing avoidable transfusion, optimizing anemia management, minimizing blood loss, and improving appropriate blood utilization across African healthcare systems.

## Methods

### Review design

This manuscript was developed as a narrative review examining restrictive vs. liberal red-cell transfusion strategies, with particular emphasis on clinical outcomes, blood-resource stewardship, and applicability within African healthcare systems. The review was designed to integrate evidence from randomized controlled trials, systematic reviews and meta-analyses, observational studies, clinical practice guidelines, consensus statements, transfusion-service reports, and relevant African policy and health-system literature. It was not designed as a systematic review or meta-analysis, and therefore did not follow a formal PRISMA systematic-review protocol.

## Literature search strategy

A structured literature search was undertaken to identify relevant publications addressing red-cell transfusion thresholds, restrictive and liberal transfusion strategies, patient blood management, transfusion outcomes, blood utilization, and African transfusion practice. Searches were conducted using major biomedical and multidisciplinary databases, including PubMed/MEDLINE, Scopus, Web of Science, and Google Scholar, supplemented by searches of relevant international and African transfusion, public-health, and professional organization websites.

The principal search concepts included combinations of terms such as:

“restrictive transfusion” OR “restrictive blood transfusion” OR “restrictive red blood cell transfusion” OR “liberal transfusion” OR “liberal blood transfusion” OR “liberal red blood cell transfusion” OR “transfusion threshold” OR “hemoglobin threshold” OR “red blood cell transfusion” OR “patient blood management”

These terms were combined with:

“Africa” OR “sub-Saharan Africa” OR “African countries” OR “low-resource settings” OR “resource-limited settings” OR “blood availability” OR “blood shortage” OR “transfusion services”

Additional disease- and population-specific searches incorporated terms related to critical illness, surgery, trauma, obstetric hemorrhage, pediatric anemia, malaria, sickle cell disease, hematologic malignancies, myelodysplastic syndromes, chronic transfusion, cardiovascular disease, and oncology. Reference lists of highly relevant articles, landmark randomized trials, systematic reviews, guidelines, and policy documents were also examined to identify additional publications not captured through database searches.

## Literature search period

The search prioritized contemporary literature while incorporating landmark studies necessary to establish the historical development of transfusion practice. Particular emphasis was placed on publications from the most recent 5–10 years, while older landmark randomized trials and foundational publications were retained where they directly informed current understanding of restrictive and liberal transfusion strategies. Recent African publications and transfusion-service literature were specifically sought to strengthen the regional relevance of the review.

## Selection of evidence

Because this was a narrative review, literature selection was relevance-based rather than governed by a prespecified systematic-review screening algorithm. Publications were considered relevant when they addressed one or more of the following:

1. Comparative restrictive vs. liberal red-cell transfusion strategies;
2. Hemoglobin-based transfusion thresholds;
3. Clinical outcomes associated with different transfusion strategies;
4. Patient blood management and blood conservation;
5. Transfusion safety and adverse outcomes;
6. Blood utilization and resource stewardship;
7. Transfusion practice in African countries;
8. Disease-specific transfusion requirements;
9. Challenges affecting blood availability and transfusion services in Africa; or
10. Future approaches to optimizing transfusion practice in resource-constrained settings.

Priority was given to studies with strong methodological designs, large patient populations, clinically relevant outcomes, contemporary relevance, and direct applicability to the review objectives.

## African-specific evidence

A specific component of the literature search focused on identifying evidence generated in African countries and African healthcare institutions. African studies were considered separately from international evidence during synthesis. Where African randomized evidence was unavailable or limited, high-quality international trials were used to establish the broader clinical evidence base, while African observational studies, national reports, transfusion-service publications, and policy documents were used to assess contextual applicability. This distinction was important because transfusion practice in Africa may be influenced by differences in disease epidemiology, baseline anemia, blood availability, component therapy, laboratory capacity, emergency transport, healthcare access, and clinical monitoring.

## Evidence hierarchy and critical appraisal

The identified literature was interpreted according to methodological hierarchy. Randomized controlled trials and high-quality systematic reviews/meta-analyses were given the greatest weight when assessing the comparative clinical effectiveness and safety of restrictive and liberal transfusion strategies. Observational studies were primarily used to provide real-world evidence and characterize African transfusion practices and health-system constraints. Clinical guidelines and consensus documents were used to contextualize current recommendations, while policy reports and expert literature informed discussions concerning implementation, blood availability, and resource stewardship. A formal quantitative risk-of-bias assessment was not performed because the manuscript was designed as a narrative review rather than a systematic review. Nevertheless, methodological limitations, population differences, potential confounding, generalizability, and consistency of findings were considered when interpreting the available evidence.

## Management of conflicting evidence

Where studies or guidelines differed in their recommendations, the evidence was interpreted according to patient population, clinical setting, transfusion threshold, disease severity, presence or absence of active bleeding, and methodological strength. Greater weight was given to high-quality randomized evidence for questions of efficacy and safety, while contextual African evidence was prioritized when discussing feasibility, blood availability, healthcare infrastructure, and implementation. Rather than assuming that one hemoglobin threshold is appropriate for all patients, the review considered transfusion as a context-dependent intervention in which laboratory values should be interpreted alongside symptoms, hemodynamic status, active bleeding, comorbidities, and anticipated clinical trajectory.

## Scope of the review

The review encompasses both international evidence and African-specific literature, but its principal objective is to determine how contemporary evidence concerning restrictive and liberal transfusion strategies can be appropriately interpreted and implemented in African healthcare settings. Particular attention was given to the tension between avoiding unnecessary transfusion and ensuring timely access to blood for patients with life-threatening anemia or hemorrhage. The review therefore adopts a patient-centered and health-system perspective, positioning restrictive transfusion within the broader framework of Patient Blood Management, rather than treating transfusion thresholds as isolated numerical rules.

## Methodological limitations

The narrative design allows integration of heterogeneous evidence and facilitates discussion of clinical and health-system issues that cannot readily be addressed through randomized trials alone. However, it also introduces the possibility of selection and interpretive bias because the literature was not subjected to a formal systematic-review screening process or quantitative risk-of-bias assessment. Furthermore, the relative scarcity of African randomized trials limits the ability to establish Africa-specific transfusion thresholds with the same degree of certainty available for some high-income-country populations. These limitations were explicitly considered when formulating the conclusions, and recommendations were framed as context-sensitive principles rather than universal transfusion rules.

### Burden of anemia and blood transfusion in Africa

Anemia remains a major public-health problem across Africa, disproportionately affecting children, adolescent girls, women of reproductive age, and pregnant women. Its causes are multifactorial and include iron deficiency, malaria, HIV and other infections, nutritional deficiencies, chronic inflammation, hemoglobinopathies, pregnancy-related blood loss, and chronic disease. The resulting clinical spectrum ranges from asymptomatic anemia to severe physiological compromise requiring urgent transfusion (12, 13). Blood transfusion is consequently an essential component of healthcare delivery across the continent. Major indications include severe childhood anemia, obstetric hemorrhage, trauma, surgery, cancer and hematological disorders, sickle cell disease, and severe acute or chronic anemia. However, demand for blood frequently exists within health systems characterized by variable donor recruitment, limited processing capacity, uneven geographic distribution, inadequate emergency stocks, and inconsistent access to specialized blood components (14, 15). The burden is not uniform across Africa. Tertiary hospitals in major urban centers may have substantially greater transfusion capacity than district and rural facilities, where delayed transportation, limited laboratory services, and inadequate blood inventories can compromise timely access. These disparities are particularly important during obstetric and traumatic emergencies, when waiting for a predetermined hemoglobin threshold may be inappropriate (16, 17).

Blood scarcity also makes resource stewardship an important component of transfusion medicine. Unnecessary transfusion consumes a finite resource that requires donor recruitment, infectious-disease screening, compatibility testing, storage, transportation, and clinical monitoring. Conversely, excessively restrictive practice may be unsafe when patients have active bleeding, severe symptomatic anemia, or limited physiological reserve (18, 19). The African transfusion context therefore requires a balance between availability, safety, clinical necessity, and conservation. Patient Blood Management provides an appropriate framework by emphasizing early detection and treatment of anemia, prevention and reduction of blood loss, and individualized transfusion when the expected clinical benefit outweighs transfusion-related risks (20). Much of the evidence supporting contemporary restrictive transfusion thresholds originates from non-African randomized trials. African-specific comparative evidence remains limited, and differences in disease burden, baseline anemia, healthcare infrastructure, blood availability, and access to emergency care may influence the applicability of international recommendations. This evidence gap highlights the need for African multicenter trials, national transfusion audits, hemovigilance systems, and implementation research to establish context-sensitive transfusion practices (Table 1) (21, 22).

**Table 1:** Burden of anemia and blood transfusion in Africa.

Domain	Key evidence/context	Implication for transfusion practice in Africa
Anemia burden	Anemia remains highly prevalent across Africa, particularly among children, women of reproductive age, pregnant women, and populations affected by infection and nutritional deficiencies.	High anemia prevalence creates substantial demand for diagnosis, prevention, treatment, and appropriately indicated transfusion.
Major causes of anemia	Iron deficiency, malaria, HIV, tuberculosis, nutritional deficiencies, hemoglobinopathies, chronic inflammation, pregnancy-related blood loss, and other chronic diseases contribute substantially.	Transfusion should address clinically significant anemia while underlying causes are investigated and treated whenever possible.
Severe childhood anemia	Severe anemia remains an important cause of pediatric hospitalization and mortality, particularly in malaria-endemic settings.	Pediatric transfusion decisions require disease-specific assessment rather than direct application of adult thresholds.
Maternal anemia and hemorrhage	Pregnancy-associated anemia and obstetric hemorrhage remain major contributors to maternal morbidity and mortality.	Reliable emergency blood supplies and rapid hemorrhage-control protocols are essential; transfusion should not be delayed during life-threatening bleeding.
Trauma and surgery	Trauma, emergency surgery, and perioperative bleeding generate substantial transfusion requirements, particularly at referral hospitals.	Emergency preparedness, blood conservation, appropriate component therapy, and rapid access to compatible blood are priorities.
Blood availability	Many African blood services experience periodic or persistent shortages, with substantial variation between countries and between urban and rural facilities.	Rational transfusion and improved inventory management are essential to preserve blood for patients with urgent indications.
Blood-component availability	Access to separated red cells, plasma, platelets, and other specialized products varies considerably across health systems.	Transfusion recommendations must account for local component availability and should avoid assuming high-income-country infrastructure.
Blood safety	Donor screening and blood-safety systems have improved substantially, but transfusion-transmitted infections and other transfusion risks remain important considerations.	Appropriate donor selection, testing, hemovigilance, and rational transfusion remain essential.
Transfusion practice	Transfusion thresholds and prescribing practices vary across institutions and countries, with limited standardized African evidence on restrictive vs. liberal strategies.	Locally adapted guidelines, transfusion committees, audit, and clinician education are needed.
Patient blood management	PBM integrates anemia prevention and treatment, blood-loss reduction, and optimization of physiological tolerance of anemia.	PBM provides a practical framework for reducing avoidable transfusion while maintaining patient safety and conserving scarce blood resources.
Evidence gap	Much of the strongest evidence on transfusion thresholds comes from non-African randomized trials, while African comparative studies remain limited.	African multicenter trials, transfusion audits, registries, and implementation studies are needed to establish context-specific evidence.

PBM, patient blood management.

### Physiological basis of restrictive and liberal blood transfusion strategies

The physiological rationale for red-cell transfusion is based primarily on maintaining adequate oxygen delivery to tissues. Hemoglobin concentration is an important determinant of arterial oxygen content, but tissue oxygenation also depends on oxygen saturation, cardiac output, microcirculatory function, and cellular oxygen utilization. Consequently, hemoglobin concentration alone does not provide a complete measure of transfusion need (23). A restrictive strategy accepts a lower hemoglobin concentration before transfusion, commonly using thresholds around 7–8 g/dL in stable adults. The approach assumes that many patients can compensate for moderate anemia through increased cardiac output, enhanced oxygen extraction, redistribution of blood flow, and other physiological adaptations. Its principal advantages are reduced exposure to donor blood, fewer transfusion-related complications, and conservation of scarce blood supplies. Restrictive transfusion is particularly appropriate for many stable, non-bleeding patients when there is no evidence of clinically significant tissue hypoxia. However, it should not be interpreted as a rigid requirement to withhold transfusion whenever hemoglobin is above a particular numerical threshold (24).

A liberal strategy uses a higher hemoglobin threshold, often approximately 9–10 g/dL, with the intention of maintaining greater oxygen-carrying capacity and physiological reserve. Historically, this approach was widely used in critically ill and postoperative patients. However, randomized clinical trials have generally failed to demonstrate a consistent clinical advantage of routinely maintaining higher hemoglobin concentrations in stable patients. Liberal transfusion may nevertheless be appropriate in selected circumstances, particularly when anemia is accompanied by active bleeding, significant symptoms, impaired oxygen delivery, or selected cardiovascular conditions (25). The physiological response to anemia varies according to age, chronicity of anemia, underlying disease, cardiovascular reserve, and rate of blood loss. A patient with chronic, compensated anemia may tolerate a lower hemoglobin concentration than a patient experiencing acute hemorrhage. Therefore, modern transfusion practice increasingly favors an individualized, physiology-informed approach in which hemoglobin is considered together with symptoms, hemodynamic status, active bleeding, comorbidities, and evidence of inadequate tissue perfusion. In Africa, this physiological framework must also be integrated with the realities of blood availability and healthcare access. Restrictive transfusion should mean avoidance of unnecessary transfusion, not delayed lifesaving transfusion, while liberal transfusion should be reserved for situations in which the anticipated clinical benefit justifies increased donor exposure and consumption of a scarce health resource (Table 2) (26).

**Table 2:** Physiological basis of restrictive and liberal blood transfusion strategies.

Physiological domain	Restrictive strategy	Liberal strategy	Clinical implication
Hemoglobin threshold	Generally uses a lower transfusion threshold, often around 7–8 g/dL in stable adults	Generally uses a higher threshold, often around 9–10 g/dL	Thresholds should be individualized rather than applied as rigid rules
Oxygen-carrying capacity	Accepts lower hemoglobin while relying on physiological compensation	Maintains higher hemoglobin and oxygen-carrying capacity	Higher Hb does not necessarily translate into improved clinical outcomes
Cardiac compensation	Relies on increased cardiac output and oxygen extraction	Provides greater oxygen-carrying reserve and may reduce compensatory demand	Patients with limited cardiovascular reserve may require individualized thresholds
Tissue oxygen delivery	Considered adequate when perfusion and oxygen utilization remain clinically satisfactory	Seeks to maintain a larger oxygen-delivery reserve	Symptoms and physiological evidence of inadequate oxygen delivery should complement Hb measurement
Transfusion exposure	Minimizes donor exposure	Increases donor exposure	Restrictive practice can reduce transfusion-associated complications
Circulatory burden	Lower risk of volume-related complications	Greater risk with unnecessary or repeated transfusion	Particularly relevant in older adults and patients with cardiac or renal dysfunction
Physiological adaptation to anemia	Allows endogenous mechanisms such as increased cardiac output and oxygen extraction	Reduces the need for compensatory mechanisms	Adaptation varies with age, disease, severity, and rate of anemia
Blood-resource utilization	Conserves scarce blood supplies	Requires greater blood consumption	Particularly important where blood availability is limited
Active hemorrhage	Hb threshold alone is inappropriate during ongoing major bleeding	Higher transfusion requirements may be necessary	Hemorrhage control and physiological status take priority over numerical thresholds
Overall principle	Transfuse when the expected clinical benefit outweighs the risks	Maintain a higher Hb reserve through earlier transfusion	Modern practice generally favors individualized, evidence-based transfusion within Patient Blood Management

Hb, hemoglobin; PBM, patient blood management.

### Restrictive vs. liberal transfusion in special populations

The optimal transfusion strategy varies considerably among patient populations. Although restrictive transfusion is generally supported for stable, non-bleeding patients, the evidence cannot be universally extrapolated to patients with active hemorrhage, acute cardiovascular disease, severe pediatric anemia, or chronic transfusion requirements. Decisions should integrate hemoglobin concentration with symptoms, physiological status, disease severity, and anticipated blood loss (27). Randomized trials such as TRICC and TRISS support restrictive transfusion, generally around 7 g/dL, in many stable critically ill adults. Liberal transfusion has not consistently improved mortality or major clinical outcomes. However, patients with active bleeding, severe hypoxemia, symptomatic anemia, or selected cardiovascular conditions require individualized assessment (28). Patients with cardiovascular disease may have reduced tolerance of anemia because of limited oxygen-delivery reserve. Although restrictive strategies remain appropriate for many stable patients, uncertainty persists in acute myocardial infarction and active myocardial ischemia. Higher transfusion thresholds may therefore be considered when clinical evidence suggests inadequate myocardial oxygen delivery (29).

For stable postoperative and perioperative patients, restrictive strategies generally reduce transfusion exposure without compromising major outcomes. However, active surgical bleeding requires immediate hemorrhage control and appropriate transfusion support rather than waiting for a predetermined hemoglobin concentration. Preoperative anemia identification and treatment are important components of PBM (30). Trauma represents a fundamentally different clinical scenario because hemoglobin may initially underestimate acute blood loss. During major hemorrhage, transfusion decisions should be guided by ongoing blood loss, hemodynamic status, coagulation abnormalities, and physiological response, rather than a fixed hemoglobin threshold. Rapid access to appropriate blood components and effective hemorrhage control are essential (4). In obstetric emergencies, particularly postpartum hemorrhage, transfusion should not be delayed while awaiting laboratory confirmation when clinically significant bleeding and physiological compromise are evident. After hemorrhage is controlled, restrictive and symptom-guided transfusion may be appropriate for residual anemia. African settings require particular attention to emergency blood stocks, transportation, and rapid access to compatible blood (31). Restrictive strategies can be appropriate for stable critically ill children, but pediatric transfusion thresholds should not simply be extrapolated from adult evidence. Age, weight, severity of anemia, oxygenation, cardiovascular status, active bleeding, and underlying disease should guide decisions (32).

Severe malaria is an important cause of transfusion in African children. Management should distinguish malaria-associated severe anemia from other causes of pediatric anemia. Transfusion should be guided by clinical severity and established disease-specific recommendations, with careful attention to volume and the risk of circulatory complications (30). Transfusion in sickle cell disease requires disease-specific indications rather than conventional restrictive-vs.-liberal thresholds alone. Simple or exchange transfusion may be indicated for selected acute or chronic complications. Repeated transfusion requires attention to alloimmunization, iron overload, delayed hemolytic reactions, and antigen matching, where feasible (4). Patients with leukemia, lymphoma, or other hematologic malignancies may develop anemia from marrow infiltration, chemotherapy, inflammation, bleeding, or nutritional deficiency. Transfusion should be individualized according to symptoms, bleeding risk, treatment status, comorbidities, and institutional protocols rather than relying exclusively on a universal hemoglobin threshold (31). Patients requiring repeated transfusions need a long-term strategy that balances symptom relief and quality of life against cumulative transfusion complications. Minimizing unnecessary donor exposure, monitoring for iron overload and alloimmunization, and considering disease-modifying therapies where available are essential (32). The evidence supports a restrictive strategy for many stable, non-bleeding patients, but transfusion thresholds should remain flexible in high-risk populations. In African practice, disease burden, delayed presentation, blood availability, laboratory capacity, and access to emergency care must be considered alongside individual clinical physiology. The appropriate goal is therefore not simply “restrictive” or “liberal” transfusion, but individualized transfusion that provides sufficient oxygen-carrying capacity while minimizing avoidable exposure to scarce blood resources (Tables 3, 4).

**Table 3:** Restrictive vs. liberal blood transfusion in special populations.

Special population	Restrictive approach	Liberal approach/when higher transfusion may be considered	Key clinical consideration
Critically ill adults	Generally favored in stable, non-bleeding patients, often using Hb threshold around 7 g/dL	May be considered with significant symptoms, impaired oxygen delivery, or selected comorbidities	Clinical stability and physiological status should guide decisions
Acute coronary disease	Lower thresholds may be appropriate in selected stable patients	Higher thresholds may be considered in acute myocardial ischemia or persistent symptoms	Evidence remains less definitive than in general critical-care populations
Perioperative patients	Generally appropriate for stable patients without active bleeding	Consider when symptomatic anemia, significant cardiovascular disease, or ongoing blood loss is present	Preoperative anemia management and blood-loss prevention are essential
Trauma/major hemorrhage	Hb threshold should not delay resuscitation	Early transfusion may be required during uncontrolled major bleeding	Hemorrhage control and balanced resuscitation take priority over Hb thresholds
Obstetric hemorrhage	Appropriate for stable postpartum anemia after bleeding control	Immediate transfusion may be required during severe or ongoing hemorrhage	Decisions should incorporate blood loss, hemodynamics, coagulopathy, and clinical response
Children	Restrictive strategies may be appropriate in stable critically ill children	Higher or immediate transfusion may be required with severe symptomatic anemia, hypoxemia, or active bleeding	Pediatric thresholds should not be directly extrapolated from adults
Severe malaria-associated anemia	Individualized according to severity and clinical condition	Transfusion may be indicated with severe anemia and clinical compromise	Malaria-related anemia requires disease-specific pediatric and adult assessment
Sickle cell disease	Avoid unnecessary transfusion; use disease-specific indications	Simple or exchange transfusion may be required for selected acute or chronic complications	Consider alloimmunization, iron overload, antigen matching, and disease-modifying therapy
Hematologic malignancies	Restrictive, symptom-guided transfusion may reduce unnecessary exposure	Higher support may be needed with active bleeding, symptomatic anemia, or selected treatment-related complications	Decisions should consider disease status, treatment, bleeding risk, and transfusion history
Myelodysplastic syndromes/chronic transfusion dependence	Avoid unnecessary exposure while maintaining clinically acceptable Hb and quality of life	Higher Hb may be appropriate for persistent symptoms or individual functional requirements	Minimize cumulative transfusion burden and monitor iron overload/alloimmunization
Renal/cardiac disease	Conservative transfusion can reduce volume-related complications	Individualized higher thresholds may be considered when anemia causes ischemic or symptomatic compromise	Balance oxygen delivery against transfusion-associated circulatory overload
Stable chronic anemia	Generally preferred when anemia is asymptomatic and treatable causes can be corrected	Consider when severe symptoms or inadequate physiological compensation occurs	Treat the underlying cause rather than using transfusion as routine anemia therapy

Hb, hemoglobin.

**Table 4:** Major evidence comparing restrictive and liberal red-cell transfusion strategies.

Study/evidence	Design and population	Restrictive strategy	Liberal strategy	Principal outcomes	Major finding and relevance to Africa
TRICC	Multicenter RCT; critically ill adults	Hb trigger ∼7 g/dL; target 7–9 g/dL	Hb trigger ∼10 g/dL; target 10–12 g/dL	30-day mortality, organ dysfunction, transfusion exposure	Restrictive transfusion was generally safe and reduced blood use. Provides foundational evidence for stable critically ill patients, although African representation was limited.
TRISS	Multicenter RCT; adults with septic shock	Hb trigger 7 g/dL	Hb trigger 9 g/dL	90-day mortality, ischemic events, adverse events, transfusion exposure	No mortality benefit from the liberal strategy; restrictive transfusion substantially reduced transfusion exposure. Relevant to resource-constrained critical-care settings.
FOCUS	RCT; older adults after hip-fracture surgery with cardiovascular risk	Hb trigger 8 g/dL or symptoms	Hb maintained ≥10 g/dL	Mortality, walking ability, cardiovascular outcomes	Liberal transfusion did not improve functional recovery or mortality. Supports restrictive practice in stable perioperative patients.
TRIPICU	Multicenter RCT; critically ill children	Hb trigger 7 g/dL in stable children	Hb trigger 9.5 g/dL	Mortality, new or progressive organ dysfunction	Restrictive transfusion was not inferior in stable critically ill children. Caution is required when extrapolating to severe malaria or actively bleeding children.
REALITY	Multicenter RCT; adults with acute myocardial infarction and anemia	Lower Hb threshold, approximately 8 g/dL	Higher threshold, approximately 10 g/dL	Major cardiovascular events and mortality	Restrictive strategy met non-inferiority criteria at 30 days, although uncertainty remains regarding selected patients with acute coronary disease.
MINT	Large multicenter RCT; patients with acute myocardial infarction and anemia	Restrictive threshold generally 7–8 g/dL	Liberal threshold generally 10 g/dL	Death or recurrent myocardial infarction	Did not establish a statistically significant superiority of liberal transfusion, but results leave uncertainty in acute myocardial infarction and support individualized decisions.
Cochrane/systematic evidence	Systematic reviews and meta-analyses of RCTs across clinical populations	Generally 7–8 g/dL thresholds	Generally 9–10 g/dL thresholds	Mortality, morbidity, transfusion exposure	Restrictive strategies usually reduce transfusion exposure without increasing mortality or major morbidity across many populations.
International guideline evidence	Major professional guidelines and consensus recommendations	Restrictive thresholds generally favored in stable, non-bleeding adults	Higher thresholds reserved for selected clinical circumstances	Patient outcomes and transfusion safety	Supports individualized restrictive practice while recognizing exceptions such as active hemorrhage and selected cardiovascular conditions.
African observational and transfusion-service evidence	Hospital studies, national/regional reports and transfusion-service literature	Variable implementation	Variable implementation	Blood utilization, availability, anemia, transfusion practice	Highlights substantial heterogeneity in African blood availability, transfusion infrastructure and clinical practice; high-quality comparative African evidence remains limited.
Patient blood management evidence	Multidisciplinary clinical and implementation evidence	Prevention of unnecessary transfusion plus anemia and blood-loss management	Greater reliance on allogeneic transfusion	Transfusion exposure, complications, resource use and outcomes	Supports moving beyond the restrictive-vs.-liberal debate toward prevention, individualized transfusion and efficient blood-resource stewardship.

Hb, hemoglobin; RCT, randomized controlled trial; TRICC, transfusion requirements in critical care; TRISS, transfusion requirements in septic shock; FOCUS, functional outcomes in cardiovascular patients undergoing surgical hip fracture repair; TRIPICU, transfusion requirements in pediatric intensive care units; REALITY, restrictive and liberal transfusion strategy in patients with acute myocardial infarction; MINT, myocardial ischemia and transfusion.

### Blood availability and resource stewardship in Africa

Blood availability is a critical determinant of transfusion safety and clinical outcomes across Africa. Although blood services have improved in many countries, substantial disparities remain in donor recruitment, collection capacity, infectious-disease screening, component preparation, storage, transportation, and distribution. These limitations are particularly consequential in rural and district facilities, where delays in obtaining compatible blood can be substantial (33). The demand for blood is driven by obstetric hemorrhage, severe childhood anemia, trauma, surgery, malaria, sickle cell disease, cancer, hematological disorders, and other causes of acute or chronic anemia. Periodic shortages can create competition between emergency and non-emergency indications, making appropriate utilization essential (33). Blood is a finite biological resource requiring considerable infrastructure and expenditure from donation through administration. Unnecessary transfusion consumes units that could be required for patients with life-threatening hemorrhage or severe anemia. Restrictive, evidence-based transfusion can therefore contribute to resource stewardship by reducing avoidable donor exposure while maintaining clinical safety. However, conservation must not become a reason to withhold clinically indicated transfusion. In patients with active major bleeding, severe symptomatic anemia, or physiological compromise, timely transfusion remains essential regardless of resource constraints (13, 34).

Patient Blood Management (PBM) provides an integrated framework for improving blood utilization (35). Its three major components are:

1. Optimizing erythropoiesis through early detection and treatment of anemia and nutritional deficiencies.
2. Minimizing blood loss through effective surgical hemostasis, obstetric hemorrhage prevention, appropriate procedural techniques, and blood-conservation measures.
3. Optimizing physiological tolerance of anemia through individualized transfusion decisions and appropriate management of underlying disease.

Implementation of PBM can reduce unnecessary transfusion while improving the resilience of blood systems. Blood shortages may coexist with wastage resulting from inappropriate inventory management, expiration, or inadequate redistribution. Strengthening demand forecasting, inventory monitoring, regional redistribution, emergency stock management, and cold-chain systems can improve availability without requiring proportional increases in collection. Digital blood-inventory systems and coordinated regional networks may further improve visibility of available components and facilitate timely redistribution (36). Safe transfusion requires reliable hemoglobin measurement, blood grouping, compatibility testing, component quality control, and recognition of transfusion reactions. Limited laboratory infrastructure and shortages of trained transfusion professionals can contribute to inconsistent practice. Strengthening laboratory services, continuous professional development, hospital transfusion committees, standardized protocols, and hemovigilance systems should therefore accompany efforts to increase blood supply (37). Because transfusion capacity varies considerably between and within African countries, a single resource-stewardship model is unlikely to be appropriate everywhere. Strategies should be adapted to local blood availability, disease burden, healthcare level, and emergency transport capacity. Future priorities should include sustainable voluntary blood donation, improved component production, reliable emergency blood stocks, stronger inventory systems, national hemovigilance, PBM implementation, transfusion audits, and African-specific research on blood utilization (Table 5) (20, 38).

**Table 5:** Blood availability and resource stewardship in Africa.

Domain	Key challenge in Africa	Implication for transfusion practice	Priority response
Blood supply	Blood shortages and seasonal fluctuations remain important in many settings	Unnecessary transfusion can reduce availability for emergencies	Strengthen voluntary donation and demand forecasting
Geographic distribution	Blood availability varies between urban tertiary centers and rural/district facilities	Patients in remote areas may experience delays in accessing compatible blood	Improve regional blood distribution, transport, and emergency stock systems
Blood components	Availability of red cells, plasma, platelets, and specialized products varies substantially	Clinical protocols must reflect locally available components	Strengthen component preparation and appropriate inventory management
Emergency transfusion	Delays may occur because of transport, testing, crossmatching, or limited emergency stocks	Waiting for a numerical Hb threshold may be unsafe during major hemorrhage	Establish emergency blood protocols and reliable hospital blood reserves
Blood wastage	Expired or poorly managed units represent avoidable loss of a scarce resource	Inefficient inventory management can worsen shortages	Use inventory monitoring, redistribution, and demand-based stock management
Appropriate utilization	Variation in transfusion thresholds and prescribing practices can lead to unnecessary transfusion	Restrictive, evidence-based practice can conserve blood without compromising care	Implement institutional transfusion guidelines, audit, and feedback
Patient blood management	Anemia is often treated late, increasing reliance on transfusion	Prevention and treatment of anemia can reduce avoidable transfusion	Integrate PBM into national and hospital policies
Laboratory capacity	Limited access to timely Hb testing, compatibility testing, and coagulation assessment in some facilities	Delayed diagnosis and decision-making may affect transfusion safety	Strengthen laboratory infrastructure and point-of-care testing
Hemovigilance	Adverse-event reporting systems remain variable	Underreporting can limit recognition of transfusion-related harm	Establish functional hospital and national hemovigilance systems
Financial resources	Blood collection, testing, processing, storage, and distribution require substantial resources	Unnecessary transfusion increases healthcare expenditure	Link transfusion stewardship with health-economic evaluation
Clinical workforce	Variable transfusion-medicine expertise and training	Inconsistent practice may contribute to inappropriate utilization	Continuous professional development and transfusion committees
Data and forecasting	Limited real-time data on demand, utilization, and wastage in some systems	Poor forecasting can produce simultaneous shortages and wastage	Develop digital blood-inventory and utilization-monitoring systems
Regional resilience	Different African countries have markedly different transfusion capacities	A single transfusion model is unlikely to suit all settings	Develop context-specific, scalable national and regional strategies

PBM, patient blood management. Key message: In Africa, blood-resource stewardship should not mean simply reducing transfusion. It should ensure that every unit is used appropriately, safely, and where it provides meaningful clinical benefit, while simultaneously strengthening blood collection, testing, distribution, inventory management, and Patient Blood Management.

### Patient blood management (PBM) in Africa

PBM provides a comprehensive framework for improving transfusion practice by shifting the focus from the availability and administration of blood toward the prevention of avoidable transfusion and optimization of the patient's own blood resources. This approach is particularly relevant to Africa, where a high burden of anemia and hemorrhage coexists with variable blood availability, limited laboratory capacity, and unequal access to transfusion services. Rather than viewing restrictive transfusion as simply a lower hemoglobin threshold, PBM integrates anemia management, prevention of blood loss, and optimization of physiological tolerance of anemia (38). The first pillar of PBM is the optimization of erythropoiesis and correction of anemia. In many African populations, anemia is associated with iron deficiency, malaria, nutritional deficiencies, chronic infections, pregnancy, inflammation, hemoglobinopathies, and other chronic diseases. Early identification of anemia, particularly before elective surgery or delivery, provides an opportunity to treat reversible causes before transfusion becomes necessary. Appropriate iron replacement, nutritional intervention, treatment of underlying infections or chronic disease, and other evidence-based therapies can reduce the severity of anemia and decrease avoidable transfusion exposure (Table 6) (39).

**Table 6:** Major randomized trials comparing restrictive and liberal red-cell transfusion strategies.

Trial	Population/setting	Restrictive strategy	Liberal strategy	Principal outcome	Major finding
TRICC	Critically ill adults	Hb trigger 7 g/dL; target 7–9 g/dL	Hb trigger 10 g/dL; target 10–12 g/dL	30-day mortality and organ dysfunction	Restrictive strategy was generally safe and substantially reduced transfusion exposure.
TRISS	Adults with septic shock	Hb trigger 7 g/dL	Hb trigger 9 g/dL	90-day mortality and ischemic events	No mortality benefit with liberal transfusion; restrictive strategy reduced blood use.
FOCUS	Older adults after hip-fracture surgery with cardiovascular risk	Hb trigger 8 g/dL or symptoms	Hb maintained around 10 g/dL	Mortality and functional recovery	Liberal transfusion did not improve survival or walking ability.
TRIPICU	Critically ill children	Hb trigger 7 g/dL	Hb trigger 9.5 g/dL	Mortality and organ dysfunction	Restrictive transfusion was safe in stable critically ill children.
REALITY	Adults with acute myocardial infarction and anemia	Lower threshold, approximately 8 g/dL	Higher threshold, approximately 10 g/dL	Major cardiovascular events and mortality	Restrictive strategy was non-inferior at 30 days, although uncertainty remains in high-risk cardiac patients.
MINT	Adults with acute myocardial infarction and anemia	Generally 7–8 g/dL	Generally 10 g/dL	Death or recurrent myocardial infarction	No statistically significant superiority of liberal transfusion; results support individualized decisions.
Systematic reviews/meta-analyses of RCTs	Multiple hospitalized and perioperative populations	Usually 7–8 g/dL	Usually 9–10 g/dL	Mortality, morbidity and transfusion exposure	Restrictive strategies generally reduce transfusion without increasing mortality or major morbidity.

Hb, hemoglobin; TRICC, transfusion requirements in critical care; TRISS, transfusion requirements in septic shock; FOCUS, functional outcomes in cardiovascular patients undergoing surgical hip fracture repair; TRIPICU, transfusion requirements in pediatric intensive care units; REALITY, restrictive and liberal transfusion strategy in patients with acute myocardial infarction; MINT, myocardial ischemia and transfusion.

The second pillar involves minimizing blood loss. Effective hemorrhage prevention and management are particularly important in African settings because obstetric hemorrhage, trauma, surgery, and gastrointestinal bleeding can rapidly exhaust local blood supplies. Strategies include meticulous surgical hemostasis, appropriate procedural techniques, early recognition of obstetric hemorrhage, rational management of anticoagulant therapy, and use of pharmacological or other blood-conservation interventions when clinically appropriate. In major hemorrhage, however, blood conservation should never delay lifesaving resuscitation or definitive control of bleeding (40). The third pillar is optimizing the patient's physiological tolerance of anemia. This requires clinicians to recognize that hemoglobin concentration is only one determinant of oxygen delivery. Symptoms, hemodynamic stability, cardiovascular reserve, oxygenation, active bleeding, underlying disease, and the rate of hemoglobin decline should be considered alongside laboratory measurements. For stable, non-bleeding patients, this principle supports restrictive and individualized transfusion strategies. Conversely, patients with active hemorrhage, severe symptomatic anemia, or evidence of inadequate tissue oxygenation may require prompt transfusion despite having hemoglobin concentrations above conventional restrictive thresholds (41).

PBM also has an important resource-stewardship function in Africa. Blood is a finite biological resource that depends on donor recruitment, collection, infectious-disease screening, compatibility testing, processing, storage, transportation, and appropriate administration. Unnecessary transfusion therefore represents both an avoidable clinical exposure and a potentially preventable use of a scarce health resource. Appropriate transfusion practices can preserve blood for patients with urgent indications such as postpartum hemorrhage, severe pediatric anemia, trauma, major surgery, and other life-threatening conditions (42). Implementation of PBM across Africa nevertheless faces important challenges. These include inconsistent preoperative anemia screening, limited access to diagnostic testing, inadequate availability of iron and other anemia therapies in some settings, variable blood-component production, shortages of trained transfusion specialists, weak transfusion committees, limited hemovigilance, inadequate clinical audit, and disparities between tertiary hospitals and rural or district facilities. In some settings, delayed transportation and limited emergency blood stocks may further complicate implementation. These challenges mean that PBM cannot simply be imported as a high-income-country model; it must be adapted to local clinical and health-system realities (43). A practical African PBM strategy should therefore combine early anemia detection and treatment, prevention of avoidable blood loss, evidence-based transfusion thresholds, appropriate blood-component use, emergency preparedness, clinician education, transfusion auditing, and strengthened blood-inventory management. Hospital transfusion committees can play an important role by developing context-appropriate protocols, monitoring transfusion indications, reviewing adverse events, and providing feedback on blood utilization. National blood services and healthcare institutions should also collaborate to improve forecasting, distribution, emergency stock management, and surveillance of transfusion practices (Figure 1) (44). Digital technologies offer additional opportunities to strengthen PBM. Electronic transfusion records and blood-inventory systems can facilitate monitoring of transfusion indications, component utilization, wastage, adverse reactions, and patient outcomes. Decision-support tools could eventually assist clinicians in identifying patients who may benefit from transfusion while reducing unnecessary exposure. However, such technologies should be developed and validated using African data and designed for settings with variable connectivity and laboratory infrastructure.

**Figure 1:**
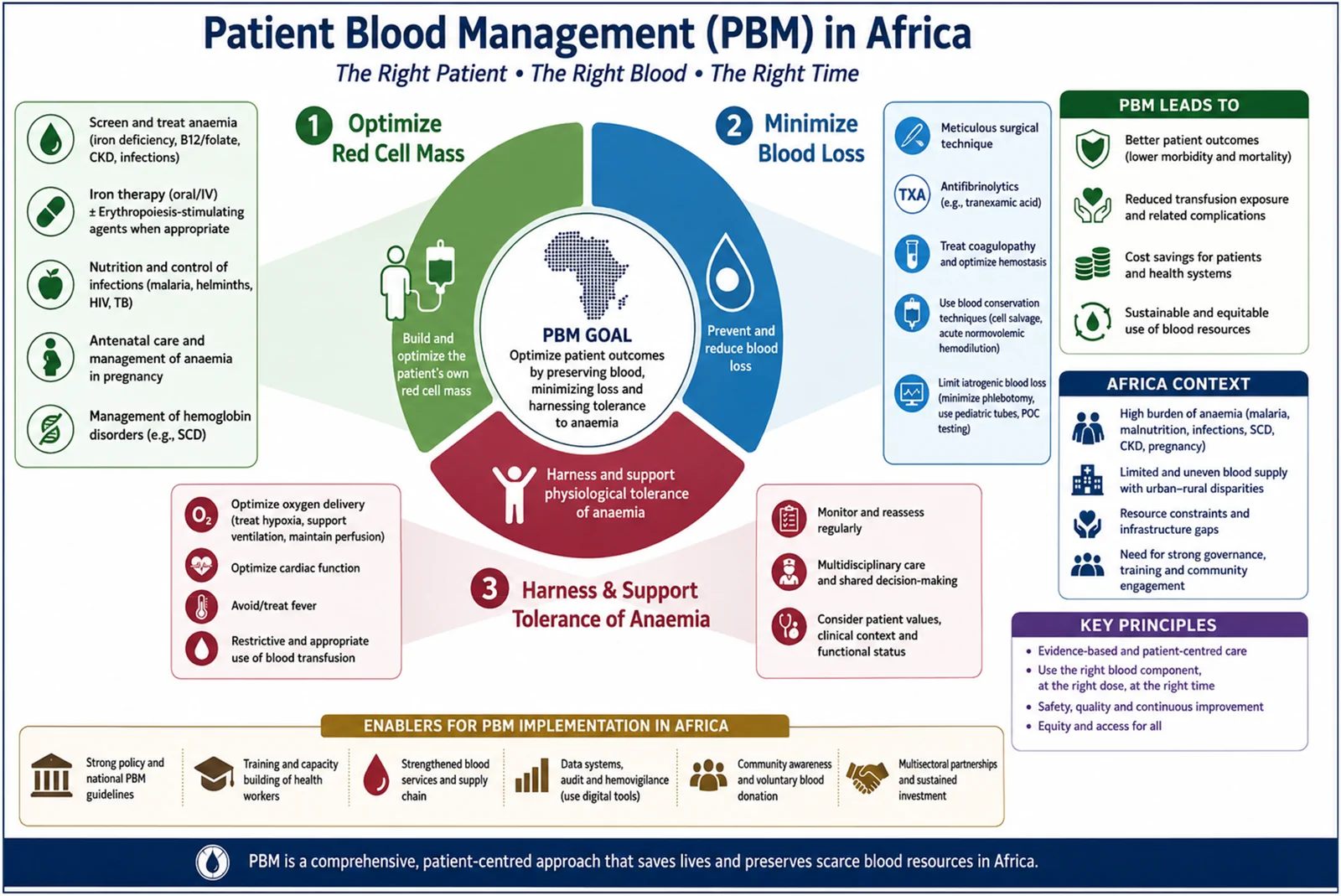
Patient blood management (PBM) in Africa.

### Emerging technologies and artificial intelligence in blood management

Emerging technologies and artificial intelligence (AI) have the potential to strengthen blood management across Africa by improving demand forecasting, inventory control, transfusion decision-making, hemovigilance, and patient blood management. Their greatest value may be in addressing persistent challenges related to unpredictable demand, blood wastage, geographic disparities, and limited specialist transfusion expertise (45). AI-driven demand forecasting can integrate historical utilization, seasonal variation, demographic information, disease patterns, surgical schedules, and emergency trends to predict future blood requirements. More accurate forecasting could help blood services reduce both shortages and unnecessary expiration of blood components. This is particularly relevant in settings where collection capacity is limited and seasonal donor fluctuations can substantially affect supply (46). Digital blood-inventory systems can provide real-time information on blood-group-specific stocks, expiry dates, component availability, and geographic distribution. Linking hospital inventories with regional or national blood-service platforms could facilitate redistribution of units before expiry and improve emergency preparedness. Such systems should be designed to function effectively despite variable internet connectivity and limited digital infrastructure (47).

AI may also support clinical transfusion decision-making. Predictive models could integrate hemoglobin concentration, bleeding status, vital signs, comorbidities, laboratory findings, transfusion history, and clinical trajectory to identify patients likely to benefit from transfusion. Decision-support systems could complement—not replace—clinical judgment and may be particularly useful where access to transfusion specialists is limited (48). In patient blood management, digital platforms can facilitate early identification of anemia, monitor preoperative hemoglobin, track iron replacement, and identify potentially avoidable transfusions. Automated audit systems could compare transfusion practice with institutional guidelines and provide feedback to clinicians, thereby supporting quality improvement (49). AI can also strengthen hemovigilance by identifying patterns of suspected transfusion reactions from electronic clinical records and laboratory data. Automated surveillance may improve detection and reporting of adverse events, although robust validation and appropriate clinical oversight are essential (50, 51). Despite these opportunities, implementation requires caution. AI systems trained predominantly on high-income-country datasets may not perform reliably in African populations because of differences in disease epidemiology, anemia causes, transfusion practices, laboratory infrastructure, and healthcare access. Poor-quality or incomplete data can also produce biased predictions. Therefore, African AI applications should prioritize local data, external validation, transparency, cybersecurity, privacy, interoperability, and human oversight (52, 53). Future research should focus on developing and validating affordable, context-sensitive digital and AI tools for blood-demand forecasting, inventory optimization, transfusion stewardship, and hemovigilance. Importantly, technological innovation should complement rather than substitute for fundamental investments in blood donation, laboratory capacity, trained personnel, reliable cold-chain systems, and equitable blood distribution (Figure 2) (54, 55).

**Figure 2:**
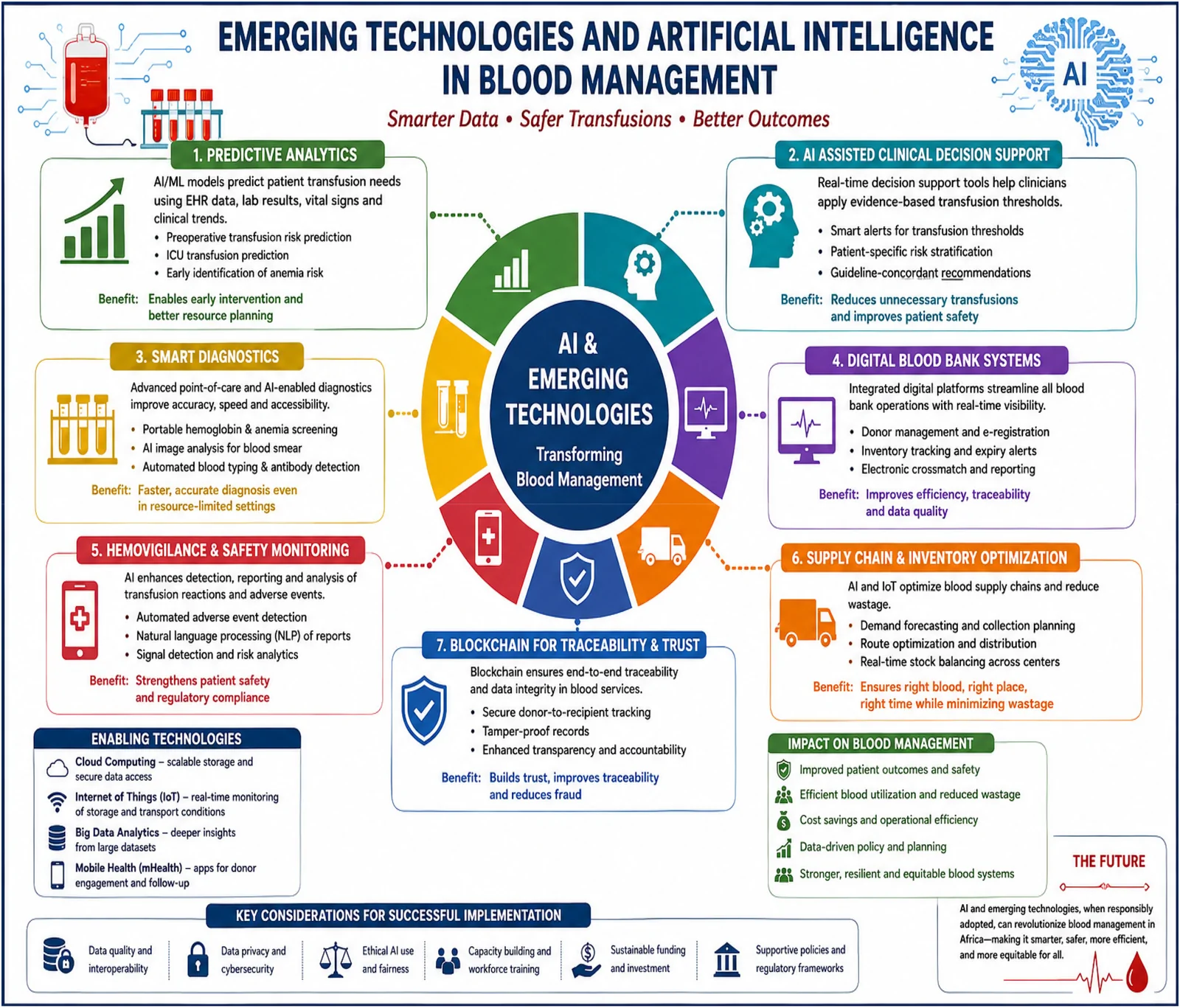
Emerging technologies and artificial intelligence in blood management.

### Implementation barriers for restrictive transfusion strategies in low-resource settings

Implementation of restrictive transfusion strategies in low-resource settings is influenced by clinical, laboratory, infrastructural, educational, and health-system factors. Although restrictive approaches can reduce unnecessary transfusion and conserve scarce blood supplies, successful implementation requires clinicians to make timely decisions based on reliable laboratory results and adequate clinical monitoring. These conditions are not consistently available across all African healthcare facilities (43). One major barrier is limited blood availability and delayed access to transfusion. In some settings, blood may not be immediately available when a patient's condition deteriorates. This can create a tendency toward precautionary or earlier transfusion, particularly when clinicians are uncertain whether blood will be obtainable later. Conversely, excessive reliance on restrictive thresholds without considering delays in blood supply could place unstable patients at risk. Effective implementation therefore requires reliable emergency blood stocks, efficient transportation, and clear escalation protocols (44). Laboratory limitations represent another important challenge. Restrictive strategies frequently depend on accurate hemoglobin measurements and, in more complex cases, assessment of coagulation, lactate, or other indicators of physiological compromise. Limited laboratory infrastructure, reagent shortages, delayed turnaround times, and inadequate access to point-of-care testing can make individualized transfusion decisions difficult. Strengthening laboratory services is therefore essential for safe implementation (Figure 3) (45).

**Figure 3:**
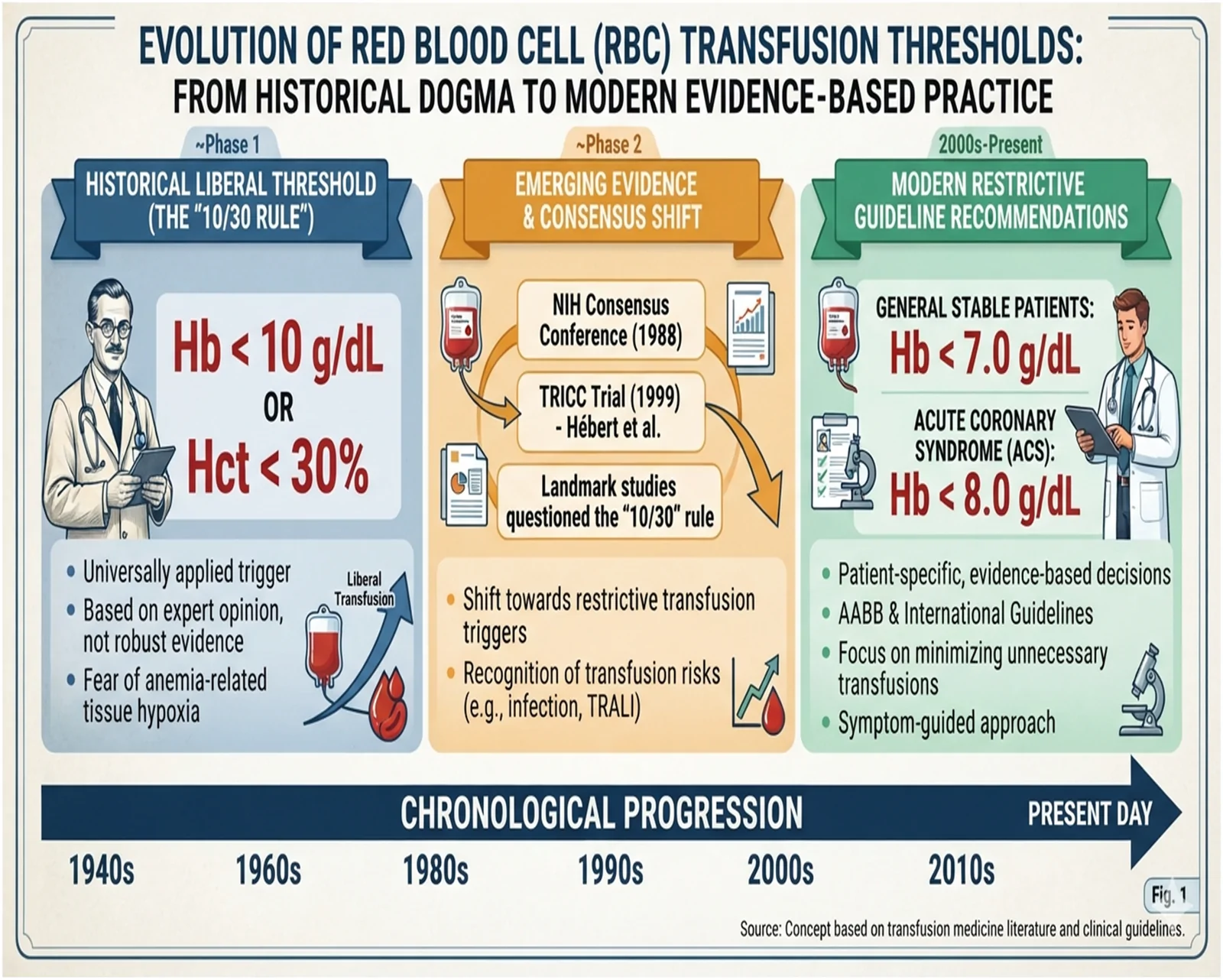
The evolution of transfusion thresholds from the historical “10/30 rule” to current guideline recommendations.

Variability in clinical knowledge and transfusion practice can also hinder adoption. Clinicians trained under traditional liberal transfusion practices may continue to use higher hemoglobin thresholds because of concerns about anemia-related complications. Inconsistent knowledge of contemporary evidence may result in substantial variation between clinicians and institutions. Regular continuing professional development, standardized protocols, and multidisciplinary transfusion education can help address this problem (46). Another barrier is the misinterpretation of restrictive transfusion as a rigid numerical rule. A restrictive strategy does not mean withholding transfusion solely because the hemoglobin concentration is above a specified threshold. Patients with active hemorrhage, severe symptomatic anemia, myocardial ischemia, or other evidence of inadequate oxygen delivery may require transfusion despite a relatively high hemoglobin concentration. Clinical protocols should therefore emphasize physiological assessment and patient-specific decision-making rather than numerical thresholds alone (47). Disease heterogeneity further complicates implementation in Africa. Severe anemia associated with malaria, sickle cell disease, pregnancy, HIV, nutritional deficiency, trauma, and hematological disorders may require different approaches. Evidence generated predominantly in high-income-country populations cannot automatically be applied to all African patients. Disease-specific guidance and locally relevant evidence are therefore necessary (Figure 4) (48).

**Figure 4:**
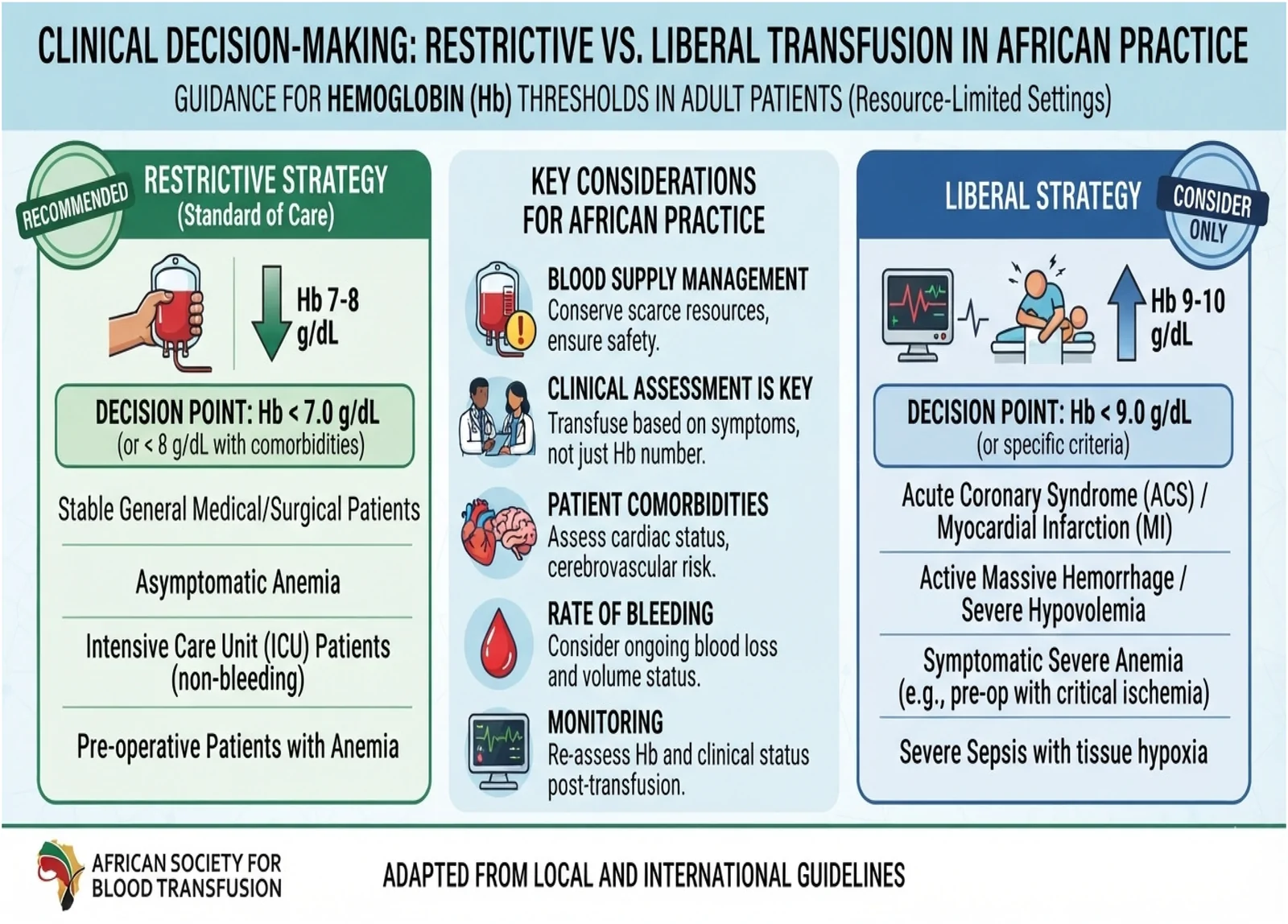
A flowchart illustrating clinical decision-making for restrictive vs. liberal transfusion in African practice.

Weak transfusion governance and limited audit systems may also reduce adherence to evidence-based practice. Hospitals without active transfusion committees, standardized indications, utilization audits, or feedback mechanisms may have difficulty identifying inappropriate transfusion. Establishing transfusion committees and routinely reviewing transfusion indications, pre- and post-transfusion hemoglobin levels, component utilization, wastage, and adverse events can improve practice (49). Limited availability of blood components presents an additional challenge. Many restrictive-transfusion trials were conducted in health systems with ready access to separated red cells, plasma, platelets, and specialized products. In some African facilities, component therapy may be limited, and whole blood may remain important. Consequently, implementation strategies must be adapted to the products and infrastructure actually available rather than directly reproducing protocols from high-resource environments (50).

Financial and logistical constraints can further limit implementation. Point-of-care devices, electronic medical records, laboratory equipment, staff training, blood-storage systems, and inventory-management technologies require sustained investment. Short-term projects without maintenance plans may produce improvements that are difficult to sustain. Restrictive transfusion programs should therefore be integrated into broader hospital quality-improvement and Patient Blood Management initiatives (51). Patient expectations and clinical culture may also influence transfusion decisions. In some circumstances, patients and families may perceive transfusion as an essential response to anemia and may be concerned when clinicians recommend observation or treatment of the underlying cause instead. Clear communication regarding the benefits and risks of transfusion and available alternatives is important for shared decision-making (52). Finally, limited African-specific evidence remains a fundamental barrier. Most major randomized trials informing restrictive transfusion thresholds were conducted outside Africa. Differences in anemia severity, infectious disease burden, nutritional status, healthcare access, blood availability, and clinical infrastructure may affect the applicability of those findings. African multicenter studies are therefore needed to establish context-specific thresholds and implementation strategies (53).

### Emerging patient blood management and blood-conservation initiatives in African healthcare settings

PBM and blood-conservation initiatives are increasingly important strategies for improving transfusion safety, reducing avoidable blood use, and strengthening the sustainability of African blood systems. Their relevance is particularly pronounced in settings where anemia and hemorrhage generate substantial transfusion demand while blood supplies, laboratory capacity, and specialized transfusion services remain unevenly distributed. Contemporary PBM approaches move beyond the simple choice between restrictive and liberal transfusion by emphasizing prevention of anemia, reduction of blood loss, optimization of physiological tolerance of anemia, and appropriate use of donor blood (54). Emerging initiatives include routine anemia screening and treatment before elective surgery and delivery, improved identification and management of iron deficiency, optimization of perioperative hemostasis, standardized obstetric hemorrhage pathways, rational use of blood components, and institutional transfusion protocols. Where appropriate and available, pharmacological and procedural blood-conservation measures can further reduce perioperative blood loss. These interventions are particularly valuable because preventing transfusion need can be more sustainable than attempting to expand blood collection alone (55).

Hospital-based transfusion committees and PBM programs can provide an important institutional structure for implementation. Such programs can establish evidence-based transfusion criteria, review inappropriate or repeated transfusions, monitor blood utilization and wastage, and provide feedback to clinical departments. Linking transfusion audits with quality-improvement activities can identify recurrent problems and allow interventions to be adapted to individual hospitals (56). Digital innovation is another emerging opportunity. Electronic transfusion records, blood-inventory platforms, computerized clinical decision support, and data-driven demand forecasting can improve monitoring of blood utilization, identify approaching expiry, support inventory redistribution, and facilitate timely clinical decisions. Artificial intelligence may eventually strengthen these systems through predictive modelling, although implementation should be preceded by validation using representative African datasets and appropriate safeguards for data quality, privacy, and clinical oversight (49). Successful PBM implementation also requires clinician training and multidisciplinary engagement. Doctors, nurses, laboratory scientists, pharmacists, blood-service personnel, and hospital administrators should understand contemporary transfusion evidence and the distinction between restrictive transfusion and inappropriate withholding of blood. Education should emphasize that hemoglobin concentration is only one component of transfusion decision-making and that active hemorrhage, severe symptomatic anemia, and physiological instability require prompt clinical intervention (57).

Local adaptation is essential. African healthcare systems differ substantially in blood availability, disease epidemiology, laboratory infrastructure, component production, referral pathways, and financial resources. Consequently, PBM protocols developed in high-income countries should not simply be transplanted without modification. National and institutional guidelines should reflect local disease patterns, available blood components, diagnostic capacity, emergency response systems, and patient needs while maintaining internationally accepted principles of transfusion safety (Figure 5) (19). Clinical audit and performance measurement are equally important for sustainability. Hospitals should monitor indicators such as transfusion rates, appropriateness of transfusion indications, pre- and post-transfusion hemoglobin levels, component utilization, blood wastage, adverse transfusion reactions, and outcomes. These data can identify gaps in practice and provide evidence for targeted interventions. National blood services can complement institutional monitoring through surveillance, hemovigilance, and coordinated inventory management (58).

**Figure 5:**
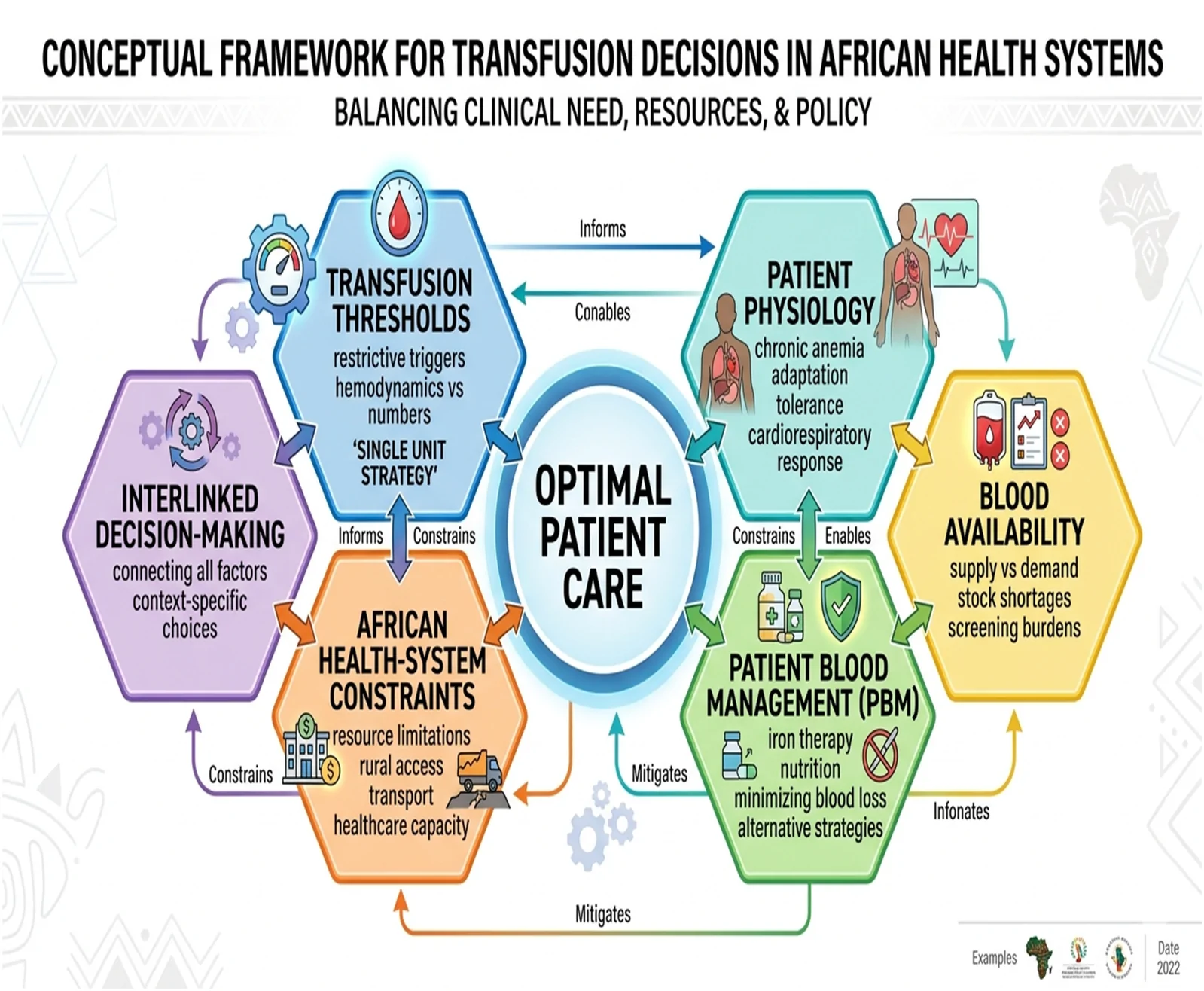
Graphical elements summarizing the relationship among transfusion thresholds, patient physiology, blood availability, patient blood management, and African health-system constraints.

Institutional leadership ultimately determines whether PBM becomes a sustainable clinical practice or remains a short-term project. Hospital leadership should provide appropriate governance structures, allocate resources for training and laboratory services, support transfusion committees, and incorporate PBM into institutional quality and patient-safety strategies. National health authorities and blood services should similarly integrate blood conservation into broader transfusion policies and health-system planning (59). The future of PBM in Africa therefore depends on a coordinated approach combining local adaptation, clinician education, regular audit, reliable data systems, institutional leadership, and sustained investment in blood-service infrastructure. The objective is not simply to reduce the number of transfusions, but to ensure that each transfusion is clinically justified, safely administered, and directed toward patients most likely to benefit. Properly implemented, PBM can simultaneously improve patient outcomes, conserve scarce blood resources, and strengthen the resilience of African transfusion systems.

### Future directions

The future of transfusion medicine in Africa should move beyond the traditional restrictive-vs.-liberal debate toward evidence-based, patient-centered, and context-adapted Patient Blood Management (PBM). Although international randomized trials provide strong evidence supporting restrictive transfusion for many stable patients, the limited representation of African populations creates uncertainty regarding the optimal application of these strategies across diverse African healthcare systems. A major priority is the development of multicenter African clinical trials comparing transfusion strategies across populations that are highly relevant to the continent, including children with severe malaria-associated anemia, pregnant women with obstetric hemorrhage, trauma patients, individuals with sickle cell disease, critically ill patients, and those with hematological malignancies. Such studies should evaluate not only mortality but also morbidity, functional recovery, transfusion reactions, blood utilization, quality of life, and cost-effectiveness. Future research should also establish African transfusion registries and national transfusion databases capable of capturing indications for transfusion, hemoglobin thresholds, blood-component utilization, shortages, wastage, adverse reactions, and patient outcomes. Reliable longitudinal data would provide a stronger basis for developing context-specific clinical guidelines and identifying variations in practice between countries and healthcare levels (43).

Another priority is the integration and scale-up of PBM programs. Hospitals should develop locally appropriate protocols for anemia detection and treatment, perioperative blood conservation, hemorrhage management, and evidence-based transfusion. These programs should be supported by transfusion committees, regular clinical audits, feedback mechanisms, and measurable performance indicators. Importantly, PBM implementation should be adapted to available resources rather than relying exclusively on models developed in high-income settings. Strengthening blood-service infrastructure remains fundamental. Future investments should improve voluntary donor recruitment, component preparation, infectious-disease screening, cold-chain systems, emergency blood reserves, transportation, and equitable distribution between urban and rural facilities. Better inventory management and regional redistribution systems could reduce both shortages and avoidable wastage (60–62).

Digital technologies and artificial intelligence offer additional opportunities. Predictive models could support blood-demand forecasting, inventory optimization, identification of potentially inappropriate transfusions, and early recognition of transfusion reactions. However, these technologies should be developed and validated using African datasets and implemented with appropriate safeguards for data quality, privacy, cybersecurity, transparency, and clinical oversight. Greater emphasis should also be placed on education and workforce development. Clinicians, nurses, laboratory professionals, blood-service personnel, and hospital administrators require continuing education in contemporary transfusion evidence, PBM, hemovigilance, and blood conservation. Incorporating transfusion medicine and PBM into undergraduate and postgraduate health-professional training could promote more consistent practice. Future guidelines should increasingly emphasize individualized transfusion decisions rather than rigid hemoglobin thresholds. Clinical assessment should incorporate symptoms, hemodynamic status, active bleeding, cardiovascular reserve, underlying disease, rate of hemoglobin decline, and availability of alternative treatments. This approach is particularly important in African settings where delayed presentation and limited emergency resources can substantially alter the risk-benefit balance. Finally, future research should examine the economic and health-system consequences of transfusion strategies. Cost-effectiveness analyses comparing restrictive transfusion, PBM interventions, expanded blood-supply systems, and digital inventory solutions could help policymakers prioritize limited resources (Figure 6) (61).

**Figure 6:**
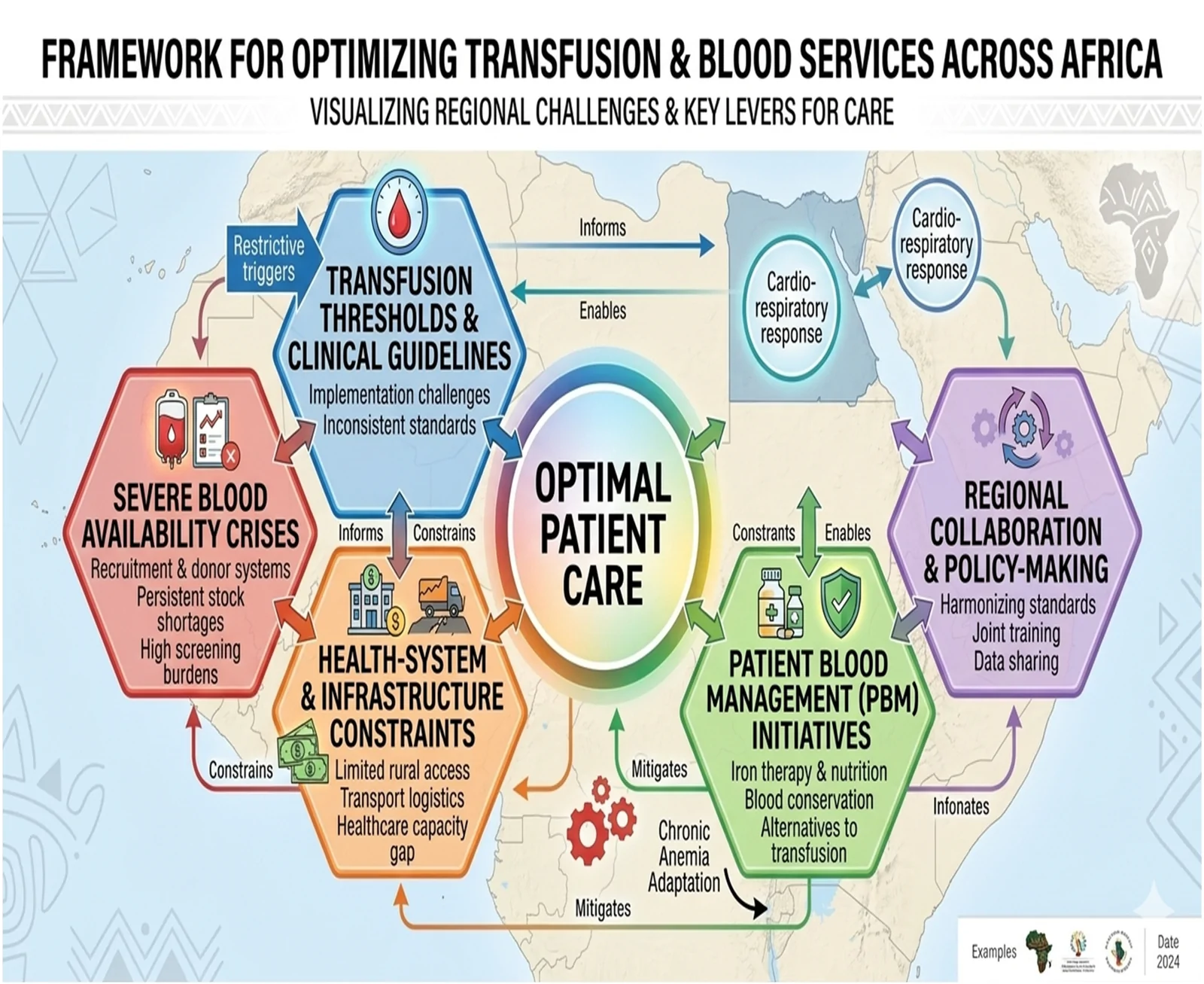
African showing regional challenges in blood availability and transfusion services.

## Conclusion

Restrictive transfusion is generally appropriate for many stable, non-bleeding adults, reducing blood exposure without compromising major outcomes. However, it should not be applied as a universal numerical rule. Patients with active hemorrhage, acute myocardial infarction, acute brain injury, severe pediatric anemia, sickle cell disease, and other high-risk conditions require individualized, disease-specific assessment. In Africa, transfusion decisions must account for the continent's substantial anemia and hemorrhage burden alongside marked variation in blood availability, laboratory capacity, emergency infrastructure, and healthcare access. Because African randomized evidence remains limited, international trial findings should be contextualized rather than directly extrapolated. PBM provides an appropriate framework by emphasizing anemia prevention and treatment, reduction of blood loss, physiological assessment, and judicious use of donor blood. Successful implementation requires local adaptation, clinician training, institutional leadership, transfusion audit, hemovigilance, and reliable blood-inventory systems. Future priorities include multicenter African transfusion-threshold trials, national transfusion registries, PBM implementation research, and validated digital technologies. Ultimately, the goal is not simply to transfuse less or more, but to provide the right blood component to the right patient at the right time, balancing clinical benefit, safety, and stewardship of scarce blood resources.
